# Meeting of the Amer. Med. Association

**Published:** 1852-03

**Authors:** P. Clairborne Gooch

**Affiliations:** One of the Secretaries, Bank Street, Richmond, Va.


ARTICLE IV.

*Meeting of the American Medical Association.*

The fifth annual meeting of the American Medical Association will be held at Richmond, Va., on Tuesday, May 14th, 1852.

All secretaries of societies, and of other bodies entitled to representation in this association, are requested to forward to the undersigned correct lists of the respective delegations as soon as they may be appointed.

The following is an extract from Art. II. of the constitution:

"Each local society shall have the privilege of sending to the association one delegate for every ten of its regular resident members, and one for every additional fraction of more than half of this number. The faculty of every regularly constituted medical college or chartered school of medicine shall have the privilege of sending two delegates. The professional staff of every chartered or municipal hospital containing a hundred inmates or more, shall have the privilege of sending two delegates; and every other permanently organized medical institution of good standing shall have the privilege of sending one delegate."

The medical press of the United States is respectfully requested to copy.

P. CLAIRBORNE GOOCH,
*One of the Secretaries,*
*Bank Street, Richmond, Va.*

